# Study on the value of gastrin 17 and aldehyde dehydrogenase 1 in gastric juice for early diagnosis in gastric cancer

**DOI:** 10.3389/fonc.2025.1521868

**Published:** 2025-01-31

**Authors:** Yanfang Wang, Hui Liu, Xiang Li, Juan Jin, Bo Yan

**Affiliations:** ^1^ Department of Gastroenterology, The Second People’s Hospital of Hefei, Bengbu Medical University, Hefei, Anhui, China; ^2^ Department of Gastroenterology, The Second People’s Hospital of Hefei, Hefei, Anhui, China

**Keywords:** gastric cancer, acetaldehyde dehydrogenase 1, gastrin 17, gastric juice, precancerous disease

## Abstract

**Objective:**

To investigate the diagnostic utility of aldehyde dehydrogenase 1 (ALDH1) and gastrin 17 (G-17) levels in the gastric juice of patients with gastric cancer and to track changes in the levels of these markers in the gastric juice of these patients.

**Methods:**

126 individuals were diagnosed via gastroscopy. This trial includes gastric mucosal histology and gastroscopy performed at Hefei Second People’s Hospital between March 2023 and March 2024. On the basis of the results of gastroscopy and gastric mucosal histology, all the participants were categorized into three groups: 30 patients with gastric cancer (GC), 34 patients with gastric ulcer (GU) and 62 patients with gastritis. Thirty patients with chronic nonatrophic gastritis were chosen from the physical examination center, and thirty-two patients with chronic atrophic gastritis (CAG) composed the gastritis group. As the control group, they were chosen at the physical testing center. An enzyme-related immunosorbent assay (ELISA) was used to quantify the levels of G-17 and ALDH1 in each group. A correlation study was performed on the levels of ALDH1 and G-17 in the gastric juice. By using binomial logistic regression analysis, the impact of G-17 and ALDH1 in gastric juice on the incidence of gastric cancer was examined. To illustrate the predictive usefulness of G-17 and ALDH1 in gastric juice for GC diagnosis, a receiver operating characteristic (ROC) curve was generated.

**Results:**

The gastric juice of the gastric cancer group had higher levels of ALDH1 and G-17 than did the gastric juice of the gastritis group and GU group (*P* < 0.05). The levels of ALDH1 and G-17 in gastric juice of gastric ulcer group were higher than those of atrophic gastritis group and control group (*P* < 0.05). The levels of ALDH1 and G-17 in gastric juice of atrophic gastritis group were statistically significant compared with those of control group (*P* < 0.05). The development of gastric cancer was significantly influenced by elevated levels of ALDH1 and G-17 in gastric juice (OR= 1.095, 1.018; both *P <*0.05); the AUCs for the combined diagnosis of gastric cancer and elevated levels of G-17 and ALDH1 in gastric juice were 0.792, 0.757, and 0.695, respectively.

**Conclusion:**

The development of gastric cancer is influenced by increased levels of ALDH1 and G-17 in gastric juice. The diagnosis of gastric cancer may be made more accurately by combining the two gastric fluid markers, which can be found in gastric juice.

## 1 Introduction

The term “gastric cancer” describes malignant tumors that arise from highly invasive epithelial cells of the gastric mucosa. It is a frequent malignant tumor of the digestive system with a high death rate and incidence rate, posing a major risk to public health. According to estimates, China has a 33.14 incidence rate per 10 million people and a 24.34 fatality rate per 10 million people for gastric cancer (1). Thus, research on the causes, progression, and metastasis of gastric cancer is crucial. Other important research includes developing novel tumor markers for the early detection, diagnosis, and treatment of gastric cancer, as well as lowering the disease fatality rate. Recently, identified tumor markers called gastrin-17 (G-17) and aldehyde dehydrogenase 1 (ALDH1) have synergistic effects on the development and growth of tumors, and they work together to drive the progression of chronic atrophic gastritis to gastric cancer (2, 3). One of the serological markers, G-17, is frequently used in the screening process for gastric cancer and precancer. It is closely associated with the processes of inflammation, immunity, invasion, and metastasis in gastric cancer cells. These processes can activate multiple signaling pathways, promote certain oncogenes, exert antiapoptotic and anti-inflammatory effects, and induce the secretion of gastric acid, all of which can contribute to the development of tumors (4). In addition to participating in gene expression and tissue differentiation in tissues, ALDH1 is a crucial marker of stem cell-like cells that affects drug resistance, invasion, metastasis, proliferation, invasion, and prognosis in cancer (5). When diagnosing gastric cancer, simultaneous findings of the two are

crucial. The development of a molecular testing method called gastric juice evaluation has high clinical relevance in gastric-related disorders and can help elucidate the secretory function of the gastric. In this study, we analyzed the changes of G-17 and ALDH1 levels in gastric juice of 126 patients treated at the Second People’s Hospital of Hefei from March 2023 to March 2024, and explored the diagnostic value of ALDH1 and G-17 levels in gastric juice for gastric cancer and their potential role in the occurrence and development of gastric cancer. We also investigated the potential clinical utility of combined detection.

## 2 Information and methodology

### 2.1 Clinical data

126 patients diagnosed between March 2023 and March 2024 at the Second People’s Hospital in Hefei City underwent gastric mucosal histopathology and gastroscopy. On the basis of the findings of the gastric mucosal histopathology and gastroscopy, all study participants were split into three groups: thirty patients in the GC group, thirty-four patients in the gastric ulcer group and sixty-two patients in the gastritis group, of whom thirty patients had chronic nonatrophic gastritis and thirty-two patients with chronic atrophic gastritis. Patients with chronic nonatrophic gastritis were used as the control group.

### 2.2 Inclusion criteria

For every participant in the research were as follows (1): had not taken any particular medications during the previous week (2); the diagnosis was confirmed by gastroscopy and pathological biopsy of gastric mucosa in all groups, with the control group showing normal or mild to moderate nonatrophic gastritis and the pathological diagnosis of chronic atrophic gastritis in the CAG group showing mild to moderate atrophy and intestinal metaplasia, which is consistent with the clinical diagnostic criteria for CAG (6); and the gastric cancer group and gastric ulcer group met the diagnostic criteria of the guidelines (7, 8). Since this was a prospective study, the researchers collected discarded gastric juice from the patient’s gastroscopy and obtained the data through ELISA, which did not adversely affect the patient’s rights and health, and all patients signed the informed consent form.

### 2.3 Exclusion criteria

All research participants were disqualified for the following reasons (1): had taken many hormones, antibiotics, gastric mucosal protectants, or acid suppressants within the previous two weeks (2); had other tumor types combined or had received radiation treatment (3); had severe cardiovascular disease or combined hepatic and renal insufficiency (4); had psychiatric disorders or were unable to collaborate with patients; and (5) had gastric juice that contained refluxed bile, blood, and food residues, among other things. Every patient gave their free will and signed an informed consent form.

### 2.4 Methods

Olympus HQ260 or 260J was used for gastroscopy under fasting conditions. Once the gastroscope reached the gastric lumen, the loop device of the loop device outer sleeve tube was inserted into the biopsy channel. Five milliliters of gastric juice were removed from the mucus lake of the gastric fundus via a 20-milliliter syringe and transferred to a sterile EP tube. The supernatant was then removed and refrigerated at -80°C for measurement, and in accordance with the standard, the gastric mucosa tissue was removed and submitted for analysis after gastroscopy was performed in accordance with the standard protocol. Pathology and gastroscopy results were considered the gold standard. Using the ELISA technique, the amounts of ALDH1 and G-17 in the gastric juices of each group of research participants were consistently assessed once during a six-month period. Hefei Biohan Biotechnology Co., Ltd. supplied the G-17 kit, while Guangzhou Taylor Biotechnology Co. supplied the ALDH1 kit.

### 2.5 Statistical processing

Utilizing SPSS 26.0 software, statistical analysis was conducted; measures conforming to a normal distribution are expressed as the mean ± standard deviation. When comparing quantitative data from multiple groups, the Kruskal-Wallis rank sum test was employed when the variance was nonhomogeneous, and one-way ANOVA was utilized when the variance was homogeneous. Independent samples t tests were used to compare two groups of quantitative data that fit a normal distribution; the Mann-Whitney U test was not used; and the chi-square test was used to compare unordered multigroup and multicategorical data. Pearson’s correlation analysis was used to examine the relationships between the levels of G-17 and ALDH1 in gastric juice and the development of gastric cancer. Additionally, binomial logistic regression analysis was used to examine the impact of G-17 and ALDH1 levels in gastric juice on gastric carcinogenesis, and receiver operating characteristic (ROC) curves were generated to illustrate the predictive value of G-17 and ALDH1 levels in gastric juice for the diagnosis of GC. At P < 0.05, the difference was considered statistically significant.

## 3 Results

### 3.1 Comparison of general information among the four groups

A total of 126 patients were included in this experiment, and they were divided into four groups according to the results of gastroscopy and pathology. According to the results of statistical analysis, there were no significant differences in age, gender, Helicobacter pylori infection status or family history of gastric cancer between GC, CAG, GU and control groups, *P* > 0.05. (**Table 1**)

**Table 1:** Comparison of the general data among the four groups.

General information	Control Group (30 cases)	CAG Group (32 cases)	GU Group (34 cases)	GC Group (30 cases)	Statistical analysis value	*p*-Value
Age, years	58.4 ± 5.5	60.1 ± 7.5	60.6 ± 9.4	62.0 ± 8.7	*H* = 3.436	0.329
Sex (%)					*χ2 =* 0.090	0.993
Female	16 (53.3)	17 (53.1)	18 (52.9)	15 (50.0)		
Male	14 (46.7)	15 (46.9)	16 (47.1)	15 (50.0)		
H. Pylori (%)					*χ2 =* 0.632	0.889
Positive	20 (66.7)	24 (75.0)	25 (73.5)	22 (73.3)		
Negative	10 (33.3)	8 (25.0)	9 (26.5)	8 (26.7)		
Family history (%)					*χ2 =* 0.931	0.818
Positive	3 (10.0)	2 (6.3)	3 (8.8)	4 (15.4)		
Negative	27 (90.0)	30 (93.7)	31 (91.2)	26 (86.7)		

Control Group, chronic nonatrophic gastritis group; CAG Group, chronic atrophic gastritis group; GU Group, gastric ulcers group; GC Group, gastric cancer group.

### 3.2 Comparison of G-17 and ALDH1 levels in the gastric juice of the four groups

**Figure 1:**
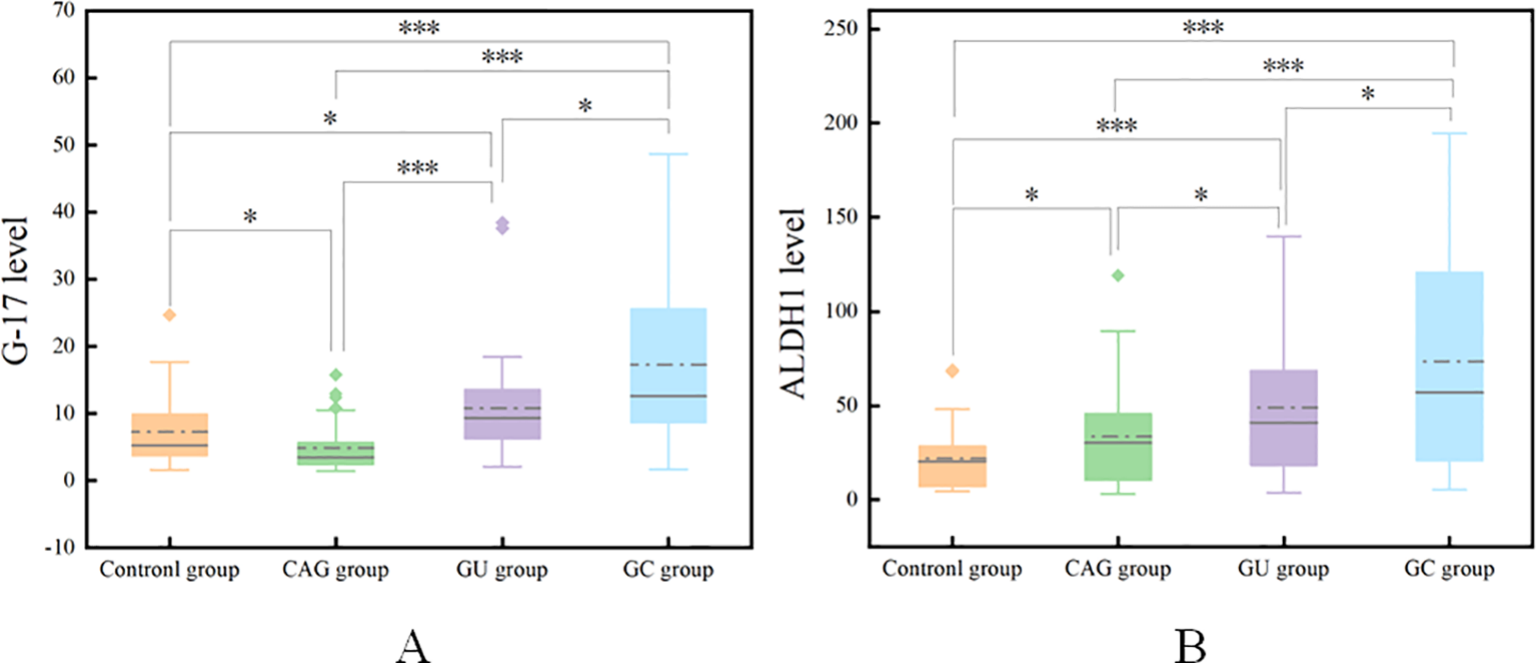
Comparison of G-17 levels in the gastric juice of the four groups **(A)**. Comparison of ALDH1 levels in the gastric juice of the four groups **(B)**. *P<0.05, ***P<0.001.

Statistical analysis showed that the contents of G-17 and ALDH1 in gastric juice of the control group were 7.26 ± 5.36 pmol/l and 21.95 ± 17.04 ng/ml, respectively, and those of the CAG group were 4.86 ± 3.72 pmol/l and 33.61 ± 27.22 ng/ml, respectively. The results were 10.78 ± 8.08pmol/l and 49.00 ± 34.43ng/ml in GU group and 17.26 ± 13.07 pmol/l and 73.56 ± 57.91 ng/ml in GC group, respectively. The levels of G-17 and ALDH1 in gastric juice of GC group were significantly higher than those of control group, GU group and CAG group (*P <*0.05). The concentrations of G-17 and ALDH1 in gastric juice of GU group were higher than those of control group and CAG group, but lower than those of GC group, with statistical significance (*P* < 0.05). (**Table 2** and **Figure 1**)

**Table 2:** Comparison of G-17 and ALDH1 levels in the gastric juice among the four groups.

	Control Group	CAG Group	GU Group	GC Group	*H*-value	*p*-Value
Number of cases (%)	30 (23.81)	32 (25.40)	34 (26.98)	30 (23.81)		
G-17 level (pmol/l)	7.26 ± 5.36	4.86 ± 3.72	10.78 ± 8.08	17.26 ± 13.07	35.650	<0.001
ALDH1 level (ng/ml)	21.95 ± 17.04	33.61 ± 27.22	49.00± 34.43	73.56 ± 57.91	21.261	<0.001

Values are presented as mean ± standard deviation (SD). G-17, gastrin-17; ALDH1, acetaldehyde dehydrogenase 1; Control Group, chronic nonatrophic gastritis group; CAG Group, chronic atrophic gastritis group; GU Group, gastric ulcers group; GC Group, gastric cancer group.

### 3.3 Comparison of G-17 and ALDH1 levels within the gastritis group

#### 3.3.1 Comparison of G-17 levels within the gastritis group

Comparison of G-17 levels within the gastritis group revealed a statistically significant difference (t = 2.056, P < 0.05) between the gastric juice levels of G-17 in the chronic atrophic gastritis group (4.86 ± 3.72 pmol/l) and those in the chronic nonatrophic gastritis group (7.26 ± 5.36 pmol/l).

#### 3.3.2 Comparison of ALDH1 levels within the gastritis groups

Comparison of ALDH1 levels within the gastritis groups revealed a statistically significant difference (t = -2.006, P < 0.05) between the chronic nonatrophic gastritis group (21.95 ± 17.04 ng/ml) and the chronic atrophic gastritis group (33.61 ± 27.22 ng/ml) in terms of ALDH1 levels in gastric juice.

### 3.4 Relationship between G-17 and ALDH1 levels in the gastric juice

Correlation analysis showed that gastric juice G-17 was positively correlated with ALDH1 levels (r=0.252, *P*<0.05). These results indicated that there was a positive linear correlation between the level of G-17 in gastric juice and the level of ALDH1 in gastric juice in the diagnosis of gastric cancer.

### 3.5 Effects of G-17 and ALDH1 levels in gastric juice on gastric carcinogenesis

Binomial Logistic regression analysis showed that the increase of G-17 level in gastric juice was an influential factor in the occurrence of gastric cancer (OR = 1.095, *P* < 0.05). Similarly, the increase of ALDH1 level in gastric juice was an influential factor in the occurrence of gastric cancer (OR = 1.018, *P* < 0.05). (**Table 3**)

**Table 3:** Effects of G-17 and ALDH1 levels in gastric juice on the occurrence of gastric cancer.

indicators	β-value	Sx value	Wald x² value	*P*-value	OR	95% CI
G-17	0.091	0.027	11.755	0.001	1.095	1.040~1.154
ALDH1	0.018	0.006	10.192	0.001	1.018	1.007~1.029

G-17, gastrin-17; ALDH1, acetaldehyde dehydrogenase 1; OR, odds ratio; CI, confidence interval

### 3.6 The value of G-17 and ALDH1 in predicting gastric cancer in gastric juice

**Figure 2:**
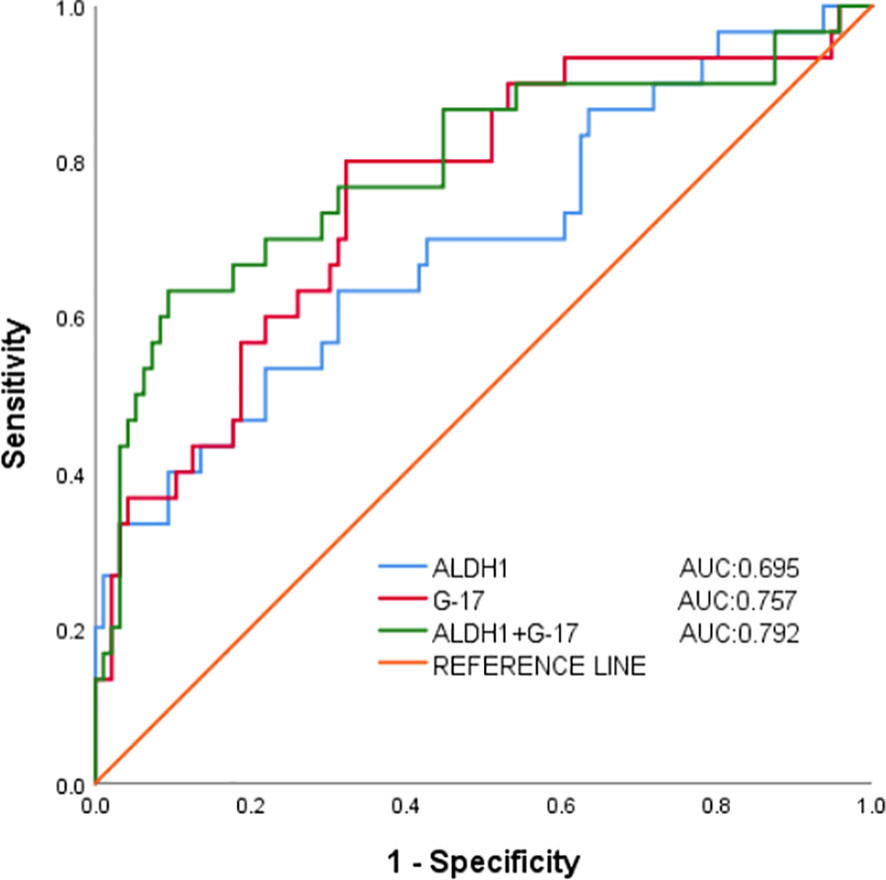
Working characterization of the ability of G-17 and ALDH1 levels in gastric juice to predict gastric cancer (ROC curve analysis).

Area under the curve (ROC) illustrating the predictive value of G-17 and ALDH1 in gastric juice for GC diagnosis. The combination diagnosis of gastric cancer had the best value, with the AUC of G-17, ALDH1, and combined diagnosis in gastric juice measuring 0.757, 0.695, and 0.792, respectively. (**Table 4** and **Figure 2**).

**Table 4:** Value of G-17 and ALDH1 levels in gastric juice for the prediction of gastric cancer.

Indicators	AUC	Youden index	cut-off value	95% CI	*p*-Value	Sensitivity	Specificity
G-17	0.757	0.477	8.316pmol/l	0.654~0.860	<0.001	0.800	0.677
ALDH1	0.695	0.320	39.506ng/ml	0.581~0.810	0.001	0.633	0.687
Combined diagnosis	0.792	0.539	–	0.686~0.898	<0.001	0.633	0.906

G-17, gastrin-17; ALDH1, acetaldehyde dehydrogenase 1; CI, confidence interval; AUC, area under curve.

### 3.7 The levels of G-17 and ALDH1 in gastric juice were used to evaluate the diagnosis of gastric cancer

Through the detection of G-17 level and ALDH1 level in gastric juice of control group, CAG group, GU group and GC group, this study proved that G-17 level and ALDH1 level in gastric juice of gastric cancer group were significantly increased, which confirmed the high diagnostic value of G-17 and ALDH1 in the diagnosis of gastric cancer. Secondly, according to the results of gastroscopy and pathology, 126 subjects in the four groups were diagnosed as GC and non-GC. Binomial logistic regression analysis suggested that G-17 level in gastric juice was an influential factor in the occurrence of gastric cancer, and the maximum Youden index calculated by ROC curve was 0.477. The optimal critical value of G-17 level in gastric juice for GC diagnosis is 8.316pmol/l, which can be used to distinguish GC from non-GC. Similarly, ALDH1 level in gastric juice is an influential factor in the occurrence of gastric cancer. The maximum Youden index calculated by ROC curve is 0.320, and the optimal critical value of G-17 level in gastric juice for the diagnosis of GC is 39.506ng/ml, which can be used to distinguish GC from non-GC. It can be concluded that the consistency rates of G-17 level and ALDH1 level in gastric juice for GC diagnosis were 70.6% and 60.3%, and Kappa value was 0.371 and 0.172, respectively (*P <*0.05). These results indicate that the diagnostic criteria of G-17 and ALDH1 in gastric juice for evaluating gastric cancer and precancerous diseases are consistent with the gold standard to a certain extent, and can be used as a reference index for early screening of gastric cancer and precancerous diseases. In addition, the positive predictive values were 80% and 63.3%, and the negative predictive values were 67.7% and 59.4%, respectively, indicating that the authenticity, reliability and predictive values of G-17and ALDH1 levels in gastric juice for screening gastric cancer are high, and have a certain degree of diagnostic value.

## 4 Discussion

With respect to incidence and mortality rates, gastric cancer is one of the most prevalent malignant tumors of the gastrointestinal tract and is among the deadliest diseases worldwide (9). Precancerous lesions such as intestinal metaplasia, heterotrophic hyperplasia, atrophic gastritis, and nonatrophic gastritis progress and eventually lead to the development, progression, and spread of gastric cancer. Because chronic atrophic gastritis affects the gastric mucosa in the digestive system, it can cause mucosal atrophy, loss of the gastric glands, intestinal epithelial chemosis, and increased oxidative stress (10). These factors are strongly linked to the progression of gastric cancer. A number of complicated and protracted elements play a role in the long-term progression of chronic atrophic gastritis to gastric cancer. Therefore, early diagnostic, therapeutic, and preventive interventions reduce the incidence of gastric cancer and precancerous disease. The digesting juice that the gastric produces, known as gastric juice, acts as the first line of defense between pathogens and the digestive tract. Research has demonstrated that the use of gastric juice as a liquid biopsy is a potential method for the noninvasive, sensitive, and precise diagnosis of early GC (11).

ALDH1 is a significant stem cell marker that has a role in controlling several genes and proteins during the genesis, development, and different phases of the disease in GC (12). ALDH1 is engaged in numerous signaling pathways that regulate tumor stem cells; plays crucial roles in cancer genesis, progression, metastasis, and chemoresistance; and can lead to new tumors via self-renewal and the generation of differentiated cancer offspring (13). ALDH1 expression is significantly higher in gastric cancer tissues than in normal tissues adjacent to cancer, and studies have demonstrated that ALDH1 is closely related to GC. Additionally, ALDH1 overexpression in gastric cancer tissues is closely correlated with clinicopathological features such as the degree of tumor differentiation, lymph node metastasis, and TNM stage and has a significant effect on patient survival time (13–15). In this study, we measured the levels of ALDH1 in the gastric juice of patients with chronic gastritis, gastric ulcers and gastric cancer. The research results show that the expression level of ALDH1 in the gastric juice of patients with chronic atrophic gastritis is higher than that of patients with chronic non-atrophic gastritis, and the expression level of ALDH1 in the gastric juice of patients with gastric ulcer is higher than that of the gastritis group. The expression level of ALDH1 in the gastric cancer group is significantly higher than that in the gastritis group and the gastric ulcer group, confirming that the expression level of ALDH1 in the gastric juice of patients in the gastric cancer group is significantly elevated. This indicates that the progression from chronic non-atrophic gastritis to gastric cancer is influenced by the level of ALDH1 in the gastric juice. Consistent with the findings of earlier studies, ALDH1 levels in gastric juice may, in some way, contribute to the development of gastric cancer. This would enable tumor stem cells (TSCs) to exert their highly invasive and metastatic potential and mediate epithelial-mesenchymal transition (EMT), which would hasten the progression and metastasis of gastric cancer (12). Additionally, these findings are consistent with the findings of earlier studies.

G-17, a peptide hormone secreted by G cells in the gastrointestinal tract, primarily stimulates the secretion of gastric acid and nourishes the mucosa of the gastrointestinal tract, maintains the structural integrity of the gastrointestinal tract, and regulates the functions of the gastrointestinal tract (16). G-17 achieves these biological effects through a cascade of signals after binding to the cholecystokinin receptor. Gastrin has a strong affinity for the upper gastrointestinal tract mucosa’s CCK-2 receptor. Once attached, it can trigger a number of signaling pathways that encourage the production of gastric acid and the growth of tumors by stimulating cell proliferation, antiapoptotic, and inflammatory responses. Recent research has demonstrated a close relationship between G-17 and the immunology, invasion, and metastasis of gastric cancer cells (4, 17). The expression level of G-17 increases dramatically in pathological conditions such as gastritis and gastric cancer (18, 19). When gastritis, gastric ulcer, gastric cancer and other diseases appeared, the expression level of G-17 increased significantly. G-17 is valuable in the prevention, screening, and diagnosis of cancer, gastric ulcers, and atrophic gastritis since screening methodologies tailored to various populations have been established, and there is a wealth of relevant research in this area (20, 21).

As a result, G-17 is crucial in the development of gastrointestinal malignancies in addition to reflecting the functional state of the gastric mucosa. In this study, the levels of G-17 in gastric juice of patients with gastric cancer, gastric ulcer and chronic gastritis were measured. The results showed that the levels of G-17 in gastric juice of patients with gastric cancer and gastric ulcer were significantly higher than those of patients with chronic gastritis. The level of G-17 in gastric juice of chronic atrophic gastritis patients was lower than that of chronic non-atrophic gastritis patients. According to statistical analysis, the level of G-17 in gastric juice of gastric cancer patients is higher than that of gastric ulcer patients and chronic gastritis patients, and the increase of G-17 level in gastric juice is an influential factor in the occurrence of gastric cancer. Therefore, the level of G-17 in gastric juice is of great value in the diagnosis and screening of gastric cancer. These findings suggest that G-17 and the CCK2 receptor may play important roles in the development of gastrointestinal cancer by activating downstream signaling pathways, such as the JAK2/STAT3, PI3K2, and PI3K2/AKT pathways, among others, which in turn promote tumor cell proliferation, migration, and invasion.

The process of developing gastric cancer is multifaceted, multistep, and interactive. Early detection, diagnosis, and treatment can lower mortality, increase life expectancy, and safeguard people’s health. Pregastric disease is a benign disease associated with gastric cancer with the possibility and risk of developing gastric cancer. Chronic atrophic gastritis and gastric ulcer are precancerous diseases of gastric cancer. By monitoring the concentration changes of G-17 and ALDH1 in gastric cancer and precancerous diseases, it can be concluded that they play an important role in gastric cancer and precancerous diseases. The levels of G-17 and ALDH1 in the gastric juice of patients with gastric cancer, gastric ulcers and chronic gastritis were found in the present study, and these levels were significantly elevated in the gastric juice of the gastric cancer group. These findings suggest that the levels of G-17 and ALDH1 have high diagnostic value in the diagnosis of gastric cancer. Correlation analysis confirmed that G-17 in gastric juice was positively correlated with ALDH1 level, and the increase of G-17 and ALDH1 levels in gastric juice was an influential factor in the development of gastric cancer. Secondly, statistical analysis showed that the positive predictive values of G-17 level and ALDH1 level in gastric juice for the diagnosis of GC were 80% and 63.3%, negative predictive values were 67.7% and 59.4%, the agreement rates were 70.6% and 60.3%, and Kappa values were 0.371 and 0.172, respectively. It indicates that the diagnostic reliability and accuracy of this experiment are high, and it can be used as a screening index for gastric cancer and provide a theoretical basis for clinical value. Additionally, the present findings demonstrated that, in comparison with separate detection, the AUC of the combined detection of G-17 and ALDH1 levels in gastric juice to diagnose gastric cancer was 0.792, suggesting that the combination detection was more valuable. This implies, at least in part, that the levels of G-17 and ALDH1 in gastric juice may be dynamically monitored to identify gastric cancer and that the combined diagnosis is more reliable and accurate than the individual diagnosis is. It can be seen that variations in G-17 and ALDH1 levels are observed in gastric tissues at every stage of the disease process, from inflammation to cancer. These changes may work in concert to facilitate the growth and spread of tumors. The location and severity of gastric mucosal lesions, as well as the diagnosis and screening of gastric cancer and precancerous lesions, may be ascertained via G-17 and ALDH1 values.

In summary, elevated levels of G-17 and ALDH1 in gastric juice are important factors in gastric carcinogenesis; the levels of G-17 and ALDH1 in the gastric juice of the gastric cancer group were abnormally highly expressed and correlated, and the value of combined detection was greater. This work also shows that gastric juice biopsy is a unique screening and diagnostic method that is very important for investigating the pathophysiology of gastric cancer and for diagnosing the disease. This study further indicates that gastric juice biopsy can be considered as a novel screening and diagnostic approach, which holds significant importance in the diagnosis and exploration of the pathogenesis of gastric cancer. Currently, research mainly focuses on the exploration of serum G-17 and ALDH1, while the study of gastric juice as a new type of liquid biopsy is still insufficient. Future studies should further explore in depth the biomarkers in gastric juice and their relationship with gastric cancer development, with the aim of developing more sensitive and specific diagnostic methods and therapeutic means for gastric cancer. In addition, this study has several limitations, such as its small sample size, and the grading and staging of gastric cancer should be further clarified to confirm the specific relationships between G17 and ALDH1 levels and the development of gastric cancer.

## Data Availability

The original contributions presented in the study are included in the article/supplementary material. Further inquiries can be directed to the corresponding author.
